# Hidden in plain sight: bias towards sick patients when sampling patients with sufficient electronic health record data for research

**DOI:** 10.1186/1472-6947-14-51

**Published:** 2014-06-11

**Authors:** Alexander Rusanov, Nicole G Weiskopf, Shuang Wang, Chunhua Weng

**Affiliations:** 1 Department of Anesthesiology, Columbia University, New York, NY, USA; 2 Department of Biomedical Informatics, Columbia University, New York, NY, USA; 3 Department of Biostatistics, School of Public Health, Columbia University, New York, NY, USA

## Abstract

**Background:**

To demonstrate that subject selection based on sufficient laboratory results and medication orders in electronic health records can be biased towards sick patients.

**Methods:**

Using electronic health record data from 10,000 patients who received anesthetic services at a major metropolitan tertiary care academic medical center, an affiliated hospital for women and children, and an affiliated urban primary care hospital, the correlation between patient health status and counts of days with laboratory results or medication orders, as indicated by the American Society of Anesthesiologists Physical Status Classification (ASA Class), was assessed with a Negative Binomial Regression model.

**Results:**

Higher ASA Class was associated with more points of data: compared to ASA Class 1 patients, ASA Class 4 patients had 5.05 times the number of days with laboratory results and 6.85 times the number of days with medication orders, controlling for age, sex, emergency status, admission type, primary diagnosis, and procedure.

**Conclusions:**

Imposing data sufficiency requirements for subject selection allows researchers to minimize missing data when reusing electronic health records for research, but introduces a bias towards the selection of sicker patients. We demonstrated the relationship between patient health and quantity of data, which may result in a systematic bias towards the selection of sicker patients for research studies and limit the external validity of research conducted using electronic health record data. Additionally, we discovered other variables (i.e., admission status, age, emergency classification, procedure, and diagnosis) that independently affect data sufficiency.

## Background

Since the passage of the Health Information Technology for Economic and Clinical Health (HITECH) Act in 2009 [1,2], there has been an increase in the rate of electronic health record (EHR) adoption. As of 2012, the rate of EHR adoption with at least basic functionality was 44.4% in non-federal acute care hospitals [3] and 39.6% in office-based physician practices [4].

The transition to EHRs has created new opportunities for research [5-7]. The secondary use of EHR data provides a more efficient and less expensive alternative to clinical trials, the current gold standard of medical research [8,9]. This is especially important in the current fiscal climate, where federal funding of medical research is becoming increasingly limited.

There are, however, potential caveats to the secondary use of EHR data [10]. EHRs suffer from data quality problems [11-13], which may affect the internal validity of retrospective studies. One of these data quality problems is insufficient data. Sufficiency can be conceptualized as a type of completeness, which is one of several categories of data quality that are relevant to EHR data reuse [14]. When EHR data are complete according to the requirements of a given task, those data can be considered to be sufficient for that task. Required data may be missing for different reasons: a data point was observed but not documented [12] or it was never observed in the first place, either because the observation was not clinically necessary or because it could not be performed. Regardless of the reason, missing data is very common in today’s EHR databases, leading to datasets that may not be sufficient for work relying on the secondary use of EHR data. Although it has been pointed out that the missing data may cause records to be “visually complete but intellectually insufficient,” [15] the causal effect of health status on data sufficiency is not the focus of this study. Instead, we focus on the correlation between the sufficiency of electronic health record data for clinical research and the underlying patient health status.

In a clinical trial a study sample is chosen based on predefined eligibility criteria. The data necessary to answer the research question is then prospectively collected for every participant. This approach ensures that all required data are present and trustworthy, but may come at the expense of limited external generalizability due to the non-representativeness of the sample [16]. In contrast, studies relying on the use of EHR data are thought to have greater external validity, having drawn their participants from actual patients receiving regular care in actual health care settings. In such studies, however, participants must be chosen based not only on the eligibility criteria but also upon the availability of sufficient data for extraction [17-19]. Example sufficiency requirements include “a sub-population who have sufficient health record data at institution *{X}* frequenting the *{X}* hospital system for routine care” and “total number of individuals that have male gender and serum creatinine 1.5 mg/dL or female gender and serum creatinine 1.3 mg/dL. The patients need to have at least 2 values over the threshold.” In a study by Green et al., of 122,270 patients satisfying eligibility criteria, only 59.7% had sufficient data [19]. Patients without the data necessary to determine eligibility or perform the analyses of interest cannot, by definition, be included in the study sample. The addition of this frequently overlooked sufficiency requirement has the potential to lead to bias in the selection of patients for inclusion in EHR based studies, which may limit their external validity.

The proportion of patients in a given population with sufficient data varies from study to study, as it depends on the research question and the necessary kinds of data required for answering that question [14,20]. We have previously demonstrated the contextual nature of EHR sufficiency, as well as the high variability of sufficient patient records in a large-scale analysis of the NewYork-Presbyterian Hospital Clinical Data Warehouse [14]. This variability is not always random; it is more likely that the pattern of data quantity is related to one or more of the variables of interest [21]. Our preliminary work indicates that the patient records containing sufficient data, i.e., those best-suited for secondary use in research, tend to belong to the sickest patients [22].

As Lee et al. point out, many studies assume that the addition of a requirement for a visit in a given time frame (visit-based sampling) produces a sample that is representative of the population from which it is derived. However, as their work demonstrates this assumption is wrong, and the imposition of just this one sufficiency requirement biases the population towards sicker and older patients [23]. Sufficient visit data is one common way patients are selected for inclusion in EHR based research. Another common sufficiency requirement is based on laboratory and/or medication data. Some studies require just the presence of a specific laboratory value or medication order while others also impose a minimum threshold for the number of each.

This paper reports an in-depth exploration of the relationship between patient illness severity and quantity of available data, as well as the potential clinical confounders of this relationship. We demonstrate that, because of the data sufficiency requirements for sampling, the cohorts being identified for research may not be representative of the broader patient population, thus compromising the external validity of research conducted using EHR data.

We hypothesized that the health records of sicker patients would be more likely to have sufficient data for research, and that this relationship would hold true when controlling for possible covariates. We also hypothesized that other patient- and procedure-related factors, such as age, sex, admission status, and the emergent nature of the procedure, would independently affect EHR data quantity.

## Methods

### Identification of a health status indicator

To study the relationship between patient health status and EHR data sufficiency, we required a measure of patient health that was not affected by data missing from the EHR. Since the most common indices of patient health and comorbidity rely on information from the EHR [24], they are influenced by missing data and are thus unsuitable for a study where data sufficiency is the dependent variable [25]. An ideal, health status assessment for our purposes would be performed prospectively via examination and testing of the patient, rather than relying upon data recorded in the EHR, but such a study would be expensive and time-consuming. The American Society of Anesthesiologists (ASA) Physical Status Classification System (Table 1) is closer to this ideal than most health status indices [26,27]. An ASA Class is a subjective assessment of illness severity determined by an anesthesia provider, using a combination of direct assessment of the patient and available information from not only the EHR but also other sources that include family members, other healthcare providers, and records from outside the institution. In cases of missing or ambiguous information, further testing may be ordered to assist in patient classification. The ASA Classification is strongly correlated with other clinical risk predictors as well as outcomes [28-31].

**Table 1:** ASA classification

**ASA class**	**Definition**
**1**	A normal healthy patient
**2**	A patient with mild systemic disease
**3**	A patient with severe systemic disease
**4**	A patient with severe systemic disease that is a constant threat to life
**5**	A moribund patient who is not expected to survive without the operation
**6**	A declared brain-dead patient whose organs are being removed for donor purposes

ASA = American Society of Anesthesiologists.

An “E” is appended to the ASA Class for emergency cases.

#### Data extraction

With approval from the Columbia University Medical Center Institutional Review Board (#AAAD1873), we queried the Department of Anesthesiology Research Database (RD) to obtain our study sample. The RD contains clinical data recorded during the provision of anesthetic services and stored in a specialized Anesthesia Information Management System (CompuRecord, Philips Healthcare, Andover, MA). The CompuRecord system is used for the documentation of all anesthetic services provided in the main operating rooms, labor and delivery floors, and ophthalmology operating suite, as well as most anesthetic services provided in the endoscopy and cardiac electrophysiology and catheterization suites, within our major metropolitan tertiary care academic medical center (Columbia University Medical Center) and two of its affiliates, a hospital for women and children (Morgan Stanley Children’s Hospital), and an urban primary care hospital (The Allen Hospital).

We queried the RD for all cases containing an International Classification of Diseases, 9th Revision (ICD-9) [32] code and a Current Procedural Terminology (CPT) [33]. We looked at the quantity of available data in the year preceding the provision of anesthetic services, and therefore excluded patients younger than one year of age. We also excluded cases where the patient had another anesthetic record in the RD in the preceding year, in order to minimize bias introduced by having multiple anesthetic services. We excluded ASA 5 and 6 cases, due to their lower incidence, and randomly selected 10,000 as our study sample from the remaining 24,073 cases.

Our primary variable of interest, patient health status, defined by ASA class, was extracted for each of the 10,000 patients. We also extracted the primary ICD-9 code, primary CPT code, age, sex, emergency classification, and admission status. Emergency classification consisted of two possible values, emergent or non-emergent, based on the presence or absence of the “E” modifier of the ASA classification. Admission status consisted of three possible values — inpatient, same day, and outpatient — as documented by the anesthesia provider. Inpatients were those who had been admitted to the hospital prior to provision of anesthesia, same day patients were those admitted to the hospital after provision of anesthesia for a period of more than 23 hours, and outpatients were those discharged from the hospital within 23 hours following completion of anesthetic services.

The Clinical Data Warehouse (CDW) contains clinical care data from Allscripts’ Sunrise Clinical Manager and ancillary services data from Cerner Millennium. We chose two kinds of data - laboratory results and medication orders – commonly used as sufficiency requirements. We queried the CDW to obtain the number of days with medication orders and the number of days with laboratory results for each patient for the year preceding the provision of anesthetic services. Two or more medication orders or laboratory results recorded on the same day would be counted once. In aligning with the concept of task-dependent data quality [14], we conceptualized data sufficiency as a count variable, as opposed to a binary variable. Consequently, each patient could have a minimum of zero and a maximum of 365 days for each of the two outcome variables.

#### Data analysis

To facilitate analysis, we grouped ICD-9 and CPT codes into 18 major categories using the Clinical Classification Software tools provided by the Healthcare Cost and Utilization Project of the Agency for Healthcare Research and Quality [34]. ICD-9 Category 15 (Certain conditions originating in the perinatal period) contained only one patient. This was deemed to be medically similar to and was thus merged into ICD-9 Category 14 (Congenital anomalies) which contained 247 patients prior to the merge. ICD-9 Categories 1, 4, 5, 12, and 18 (Infections and parasitic diseases, Diseases of the blood and blood forming organs, Mental disorders, Diseases of the skin and subcutaneous tissue, and Supplementary, respectively) contain diseases not usually treated with procedures that require anesthetic services and thus contained few patients (10, 19, 9, 83, and 112, respectively). These were merged with ICD-9 Category 16 (Symptoms, signs, and ill-defined conditions) which contained 369 patients prior to merging.

**Figure 1:**
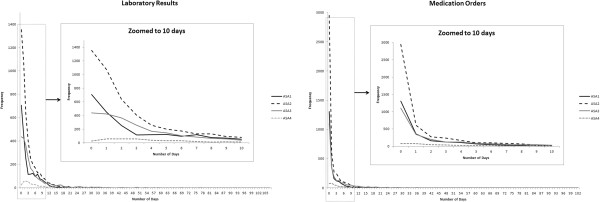
**Marginal distributions for laboratory results and medication orders. **Each curve shows the number of patients (y-axis) as a function of the number of days (x-axis) with Laboratory Results (left panel) or Medication Orders (right panel) for a given ASA Class. The insets provide a closer look at the curves in the range of 0 to 10 days.

Similar CPT categories were merged as follows: CPT Category 4 (Operations on the ear) containing 85 patients, was merged into CPT Category 5 (Operations on the nose, mouth and pharynx) containing 345 patients; CPT Category 8 (Operations on the hemic and lymphatic system) containing 106 patients, was merged into CPT Category 15 (Operations on the integumentary system) containing 336 patients; CPT Category 13 (Obstetrical procedures) containing 26 patients was merged into CPT Category 12 (Operations on the female genital organs) containing 557 patients; and CPT Category 17 (Other) containing 3 patients, was merged into CPT Category 18 (Anesthesia procedures) containing 1752 patients.In our sample 25.2% of patients had no laboratory results and 54.3% had no medication orders. Marginal distributions for laboratory results and medication orders grouped by ASA Class are shown in Figure 1.

The variations of the counts of laboratory results and medication orders are far greater than the means. To account for this over-dispersion we fit a negative binomial regression [35]. While the Poisson regression (which uses a Poisson distribution) is commonly used for analyses of count data, it does not handle over-dispersed data sets well due to the assumption that the variance of counts equals the mean. The negative binomial regression is an extension of the Poisson regression that is particularly well suited for over-dispersed count data, such as ours, where the variance is greater than the mean. In the negative binomial model, the counts Y follow a Poisson distribution (λ), where λ is a random variable with a gamma distribution. Therefore, the unconditional distribution of Y is a negative binomial.

## Results

The mean age of patients in our sample was 45.0 (SD = 23.9) and ranged from one year to 102. Sixty-one percent of our cohort was female. Most cases were non-emergent (88.8%) with more outpatients (41.6%) than same-day admissions (32.7%) or inpatients (25.6%). The most frequently occurring diagnostic categories in our dataset were Complications of pregnancy, childbirth, and puerperium (19.3%), Diseases of the digestive system (12.3%), and Neoplasms (11.1%). The most common procedure categories were Anesthesia procedures, which includes procedures for analgesia during labor and delivery (17.5%), Operations on the digestive system (16.3%), and Operations on the musculoskeletal system (13.2%). Table 2 presents descriptive statistics of counts of days with laboratory results and medication orders within subcategories.

**Table 2:** Model inputs with counts of days with laboratory results and medication orders (n = 10,000)

		**Laboratory results**		**Medication orders**	
**Variable**	**n (%)**	**Max**	**Mean (SD)**	**Max**	**Mean (SD)**
**ASA class**					
**1**	2263(22.6)	20	2.9(3.4)	21	1.3(2.2)
**2**	4779(47.8)	85	3.0(4.4)	62	1.6(3.6)
**3**	2499(25.0)	107	5.7(9.4)	102	4.1(8.5)
**4**	459(4.6)	99	9.4(13.2)	91	7.5(11.5)
**Sex**					
**Male**	3943(39.4)	99	3.4(7.4)	91	2.3(6.3)
**Female**	6057(60.6)	107	4.3(6.2)	102	2.5(5.5)
**Age (years)**					
**1-10**	911(9.1)	52	1.4(4.0)	62	1.6(4.8)
**11-20**	837(8.4)	49	2.7(4.5)	91	1.9(4.9)
**21-30**	1379(13.8)	78	5.3(6.1)	71	2.8(5.0)
**31-40**	1461(14.6)	107	5.0(6.5)	102	2.1(5.5)
**41-50**	981(9.8)	85	3.4(6.3)	43	2.0(5.0)
**51-60**	1182(11.8)	99	4.1(8.8)	76	2.9(7.7)
**61-70**	1484(14.8)	99	3.7(7.2)	76	2.5(6.2)
**71-80**	1143(11.4)	60	3.9(6.7)	63	2.6(6.0)
**81-90**	545(5.5)	60	4.7(7.3)	55	3.2(6.1)
**91-102**	77(0.8)	59	6.9(9.1)	44	5.1(7.3)
**Emergency status**					
**Non-emergent**	8883(88.8)	107	3.7(6.3)	102	2.2(5.5)
**Emergent**	1117(11.2)	99	5.5(9.2)	81	4.0(7.9)
**Admission status**					
**Outpatient**	4162(41.6)	85	2.1(4.4)	56	1.3(3.8)
**Same day**	3274(32.7)	69	3.7(4.8)	76	1.7(4.0)
**Inpatient**	2564(25.6)	107	7.2(9.9)	102	5.1(8.8)
**ICD-9 category name (number)**					
**Neoplasms (2)**	1111(11.1)	53	3.3(5.4)	62	1.8(4.8)
**Endocrine, nutritional, and metabolic & immunity disorders (3)**	211(2.1)	59	3.0(6.0)	44	1.3(4.4)
**Dz. of the nervous system and the sense organs (6)**	942(9.4)	99	1.9(6.0)	70	1.6(4.9)
**Dz. of the circulatory system (7)**	1005(10.0)	92	5.2(8.5)	81	3.9(7.4)
**Dz. of the respiratory system (8)**	368(3.7)	60	3.7(8.2)	91	3.2(9.0)
**Dz. of the digestive system (9)**	1232(12.3)	107	3.7(8.2)	102	2.7(7.3)
**Dz. of the genitourinary system (10)**	887(8.9)	92	3.9(7.1)	65	2.3(6.4)
**Complications of pregnancy, childbirth and the puerperium(11)**	1931(19.3)	54	6.3(4.4)	28	2.6(3.3)
**Dz. of the musculoskeletal system and connective tissue (13)**	767(7.7)	59	1.8(4.3)	60	1.3(4.3)
*Congenital anomalies (14)	248(2.5)	15	1.4(2.1)	34	1.1(3.2)
*Symptoms, signs and ill-defined conditions (16)	602(6.0)	99	5.1(9.0)	62	3.4(7.6)
**Injury and poisoning (17)**	696(7.0)	77	2.4(6.2)	76	2.3(5.7)
**CPT category name (number)**					
**Operations on the nervous system (1)**	460(4.6)	78	2.4(5.8)	70	1.9(6.1)
**Operations on the endocrine system (2)**	198(2.0)	21	1.7(3.2)	29	0.9(3.6)
**Operations on the eye (3)**	664(6.6)	99	1.8(5.9)	52	1.4(4.5)
*Operations on the nose, mouth, and pharynx (5)	430(4.3)	44	1.1(3.1)	36	1.1(3.1)
**Operations on the respiratory system (6)**	198(2.0)	60	6.5(10.0)	91	5.6(12.2)
**Operations on the cardiovascular system (7)**	1105(11.1)	92	5.9(9.2)	76	4.5(8.1)
**Operations on the digestive system (9)**	1625(16.3)	107	4.0(8.5)	102	2.7(7.1)
**Operations on the urinary system (10)**	533(5.3)	57	3.7(5.4)	65	1.5(4.9)
**Operations on the male genital organs (11)**	345(3.5)	49	2.5(3.8)	33	1.1(3.4)
*Operations on the female genital organs (12)	603(6.0)	28	3.0(2.9)	17	1.2(2.5)
**Operations on the musculoskeletal system (14)**	1321(13.2)	77	2.0(4.9)	67	1.7(4.8)
*Operations on the integumentary system (15)	442(4.4)	54	3.5(6.5)	53	2.4(5.6)
**Miscellaneous diagnostic and therapeutic procedures (16)**	321(3.2)	92	4.2(9.5)	81	3.1(7.8)
*Anesthesia procedures (18)	1755(17.6)	54	6.7(4.5)	52	2.8(3.5)

ASA Class = American Society of Anesthesiologists Physical Status Classification; ICD-9 = International Classification of Diseases, Ninth Revision; CPT = Current Procedural Terminology; Dz. = Diseases; * Denotes ICD and CPT categories that contain other similar categories: Congenital anomalies contains Certain conditions originating in the perinatal period; Symptoms, signs and ill-defined conditions contains Infections and parasitic diseases, Diseases of the blood and blood forming organs, Mental disorders, Diseases of the skin and subcutaneous tissue, & Supplementary; Operations on the nose, mouth and pharynx contains Operations on the ear; Operations on the female genital organs contains Obstetrical procedures; Operations on the integumentary system contains Operations on the hemic and lymphatic system; Anesthesia procedures contains Other.

Table 3 shows the effect of each variable as a whole and of each level of the primary outcome variable (ASA Class) on the estimated number of days with laboratory results and medication orders based on the parameter estimates from the negative binomial model. Effects, standard errors and 95% confidence intervals for individual variable levels are expressed as ratios comparing that level to the reference level for the variable, such that an effect of 2.0 for ASA 3 indicates that ASA 3 is estimated to have 2 times the number of days as ASA 1. These ratios were obtained by exponentiating the model regression coefficients. ASA class, subject sex, age, admission status, ICD-9 category, and CPT category were significantly associated with the counts of days with laboratory results and medication orders, while emergency status was associated only with laboratory results.

**Table 3:** Negative binomial regression

	**Laboratory results**				**Medication orders**				
**Variable**	**Overall variable effect p-value**	Ratio of expected number of days(SE)^*^	**95% Confidence interval**	**Regression p-value**	**Overall variable effect p-value**	Ratio of expected number of days(SE)^*^	**95% Confidence interval**	**Regression p-value**	
**ASA class**	<.001^†^				<.001^†^				
**1**		1.00					1.00		
**2**		1.47(1.03)	1.38 – 1.57	<.001^†^			1.74(1.05)	1.56 – 1.94	<.001^†^
**3**		3.38(1.04)	3.11 – 3.67	<.001^†^			4.78(1.07)	4.18 – 5.48	<.001^†^
**4**		5.05(1.07)	4.41 – 5.77	<.001^†^			6.85(1.12)	4.49 – 8.54	<.001^†^
**Sex**	<.001^†^					<.001^†^			
**Age**	<.001^†^					<.001^†^			
**Emergency status**	<.001^†^					0.84			
**Admission status**	<.001^†^					<.001^†^			
**ICD-9 category**	<.001^†^					<.001^†^			
**CPT category**	<.001^†^					<.001^†^			

ASA Class = American Society of Anesthesiologists Physical Status Classification; ICD-9 = International Classification of Diseases, Ninth Revision; CPT = Current Procedural Terminology; SE = Standard Error.

*The effects are ratios, obtained by exponentiating the model regression coefficients, of the expected number of days (with either laboratory results or medication orders) for each ASA Class compared to ASA 1, that is, an effect of 2.0 for ASA 3 indicates that ASA 3 is estimated to have 2 times the number of days as ASA 1.

^†^Statistically significant at the 0.05 significance level.

Our primary variable of interest, ASA class, had a significant association with the counts of days with laboratory results and medication orders. Controlling for all other variables, the estimated count of days with laboratory results for ASA 2 was 1.47 times, for ASA 3 was 3.38 times, and for ASA 4 was 5.05 times the count of days with laboratory results for ASA 1. The pairwise differences for counts of days with laboratory results between all four ASA classes were statistically significant. Similarly, the estimated count of days with medication orders for ASA 2 was 1.74 times, for ASA 3 was 4.78 times, and for ASA 4 was 6.85 times the count of days with medication orders for ASA 1. All pairwise comparisons between the four ASA classes for counts of days with medication orders were statistically significant.

## Discussion

The results of the negative binomial regression model demonstrate the relationship between patient health status and EHR data sufficiency. The less healthy the patient, as measured by ASA status, the more data that patient is likely to have, as represented by counts of days with laboratory results and medication orders, and the more likely they are to satisfy sufficiency requirements. This relationship holds true even when controlling for a number of likely confounders, including sex, age, emergent status, patient type, diagnosis, and procedure, which suggests that even within specific, well-defined cohorts, sicker patients are likely to have more data than healthier patients.

These findings highlight an important but usually overlooked problem inherent to studies using EHR data: the selection of records with sufficient data, as measured by human imposed sufficiency requirements, for research may bias the sample towards patients who are sicker than the population from which the sample is drawn. The findings from this study are consistent with previous work exploring the complex relationships among data quality, bias, and health status. In one example, Wennberg et al. used insurance claims data to demonstrate bias in comorbidity measurement by showing that Charlson Comorbity Index scores are associated with the frequency of physician visits [25], suggesting that data quality is compromised by differences in healthcare utilization. Similarly, Collins et al. identified a relationship between patient mortality and increased rates of nursing documentation, suggesting that more acutely ill patients are likely to have more thoroughly documented records [36,37]. In a study of a pneumonia severity index using EHR data, Hripcsak et al. found that the addition of cohort selection criteria that required the presence of sufficient data to make a reliable diagnosis substantially limited the sample size and significantly altered the mortality rates [38]. They note that the addition of simple sample restraints, while beneficial in their case, has the potential to significantly narrow the sample, leading to the possibility of bias.

We observed a direct correlation between severity of illness and data sufficiency in spite of the presence of sub-populations in our study sample in which this correlation should not exist: living organ donors and pregnant women with uncomplicated pregnancies presenting for management of labor and delivery. These patients tend to be healthy, but have more data in their records, resulting from laboratory testing performed as part of routine prenatal care or organ donor evaluation. Our 10,000-patient sample contained 1,802(18.0%) such patients, of whom 1,746(96.9%) were classified as ASA 1 or 2 (relatively healthy). The average number of days with laboratory results for patients in this group (6.5) is nearly double that of all other patients in the study (3.4). Despite the presence of such a large number of healthy patients with a high degree of EHR sufficiency, our original hypothesis — that sicker patients have better EHR data sufficiency for research — was confirmed. (See Additional file 1: Table S1 for results of the negative binomial model with pregnant patients and living organ donors excluded).

In addition to confirming our primary hypothesis, we discovered that many other variables are independently associated with data sufficiency. These include admission status at time of assessment, age, emergency classification of the procedure, procedure type (CPT category) and primary diagnosis type (ICD-9 category). Potential biases in these other characteristics of the study population should be considered when selecting populations based on sufficiency requirements this population is studied.

### Limitations and future directions

This study was performed primarily in a tertiary care academic medical center (though one of the included hospitals is a primary care facility) in a major metropolitan area. Consequently, many of the patients included in our analysis were likely referred from other facilities. Data might differ in a more rural, primary practice setting or in a health system where patients receive the majority of their care within that one system. A follow-up study should be performed to determine whether our results could be replicated in other clinical settings.

Since our findings are based only on data primarily collected for documentation of clinical care, we cannot definitively conclude that this same bias would exist for secondary use of data primarily collected for other purposes, such as regulatory oversight and billing. Further analysis should be performed on other data sources.

As a result of our decision to use ASA class as a measure of health status, our sample was limited to patients who had received anesthetic services. Though anesthetic services are generally provided to a wide range of patients, and one might therefore expect the relationship between record sufficiency and patient health to hold true more broadly, the generalizability of our results to other populations may be limited. A novel measure of health status that is independent of data quality but available for all patients in the EHR would provide a means to evaluate the correlation between health status and data sufficiency. Alternatively, a study that prospectively evaluates a representative sample of all patients in the EHR for health status could determine if the correlation exists in a more general population, though such a study would be costly. As in any retrospective study, it is possible that there exist covariates not controlled for in our model that account for the observed differences.

## Conclusions

In this analysis, we established the correlation between the degree of patient sickness and the sufficiency of data of their health records, for inclusion in research. This finding is important for researchers reusing EHR data. EHR-based studies sample patients based on sufficiency requirements with the aim of selecting only those records containing sufficient data to overcome the data missingness problem. This strategy turns out to introduce a hidden bias towards sick patients because sicker patient have records with a higher degree of data sufficiency and are more likely to be included in EHR-based studies; therefore, this selection process biases the study populations towards those comprised of sicker patients. The more stringent the sufficiency requirements, the sicker the resultant sample population. This is a problem unique to studies that rely on the secondary use of data initially collected for purposes other than research. Those involved in the secondary use of EHR data for research, as well as consumers of this research, should be aware of this sampling bias problem and exercise caution when applying results to real world populations.

## Competing interests

The authors declared that they have no competing interests.

## Authors’ contributions

AR and NGW carried out data extraction and analysis and wrote the manuscript together. SW performed the statistical analyses. CW identified the research question and directed the experiment. All authors read and approved the final manuscript.

## Pre-publication history

The pre-publication history for this paper can be accessed here:

http://www.biomedcentral.com/1472-6947/14/51/prepub

## Supplementary Material

Additional file 1: Table S1 Negative binomial regression excluding pregnant patients and living organ donors. Click here for file
