# Supplementary material for: Hidden in plain sight: bias towards sick patients when sampling patients with sufficient electronic health record data for research
Source: BMC Med Inform Decis Mak. 2014 Jun 11;14:51. doi: 10.1186/1472-6947-14-51 (PMC4062889; doi:10.1186/1472-6947-14-51)
Supplement: Additional file 1: Table S1 — Negative binomial regression excluding pregnant patients and living organ donors. [file 1472-6947-14-51-S1.docx]

***Additional file 1: Table S1.* Negative Binomial Regression Excluding Pregnant Patients and Living Organ Donors**

**(n=8,198)**

|  |  | **Laboratory Results** | | | | **Medication Orders** | | | |
| --- | --- | --- | --- | --- | --- | --- | --- | --- | --- |
| **Variable** | | **Overall**  **variable**  **effect**  **p-value** | **Ratio of expected number of days(SE)^*^** | **95% Confidence Interval** | **Regression p-value** | **Overall variable effect**  **p-value** | **Ratio of expected number of days(SE)^*^** | **95% Confidence Interval** | **Regression p-value** |
| **ASA Class** | | <.001† |  |  |  | <.001† |  |  |  |
|  | **1** |  | 1.00 |  |  |  | 1.00 |  |  |
|  | **2** |  | 1.86(1.05) | 1.69 – 2.03 | <.001† |  | 1.90(1.07) | 1.65 – 2.19 | <.001† |
|  | **3** |  | 4.38(1.05) | 3.96 – 4.85 | <.001† |  | 4.90(1.08) | 4.19 – 5.73 | <.001† |
|  | **4** |  | 6.34(1.08) | 5.46 – 7.40 | <.001† |  | 6.50(1.13) | 5.09 – 8.30 | <.001† |
| **Sex** | | <.001† |  |  |  | <.001† |  |  |  |
| **Emergency Status** | | <.001† |  |  |  | <.007† |  |  |  |
| **Admission Status** | | <.001† |  |  |  | <.001† |  |  |  |
| **ICD-9 Category** | | <.001† |  |  |  | <.001† |  |  |  |
| **CPT Category** | | <.001† |  |  |  | <.001† |  |  |  |

ASA Class = American Society of Anesthesiologists Physical Status Classification; ICD-9 = International Classification of Diseases, Ninth Revision; CPT = Current Procedural Terminology; SE = Standard Error

*The effects are ratios, obtained by exponentiating the model regression coefficients, of the expected number of days (with either laboratory results or medication orders) for each ASA Class compared to ASA 1, that is, an effect of 2.0 for ASA 3 indicates that ASA 3 is estimated to have 2 times the number of days as ASA 1.

†Statistically significant at the 0.05 significance level
